# Supplementary material for: Preliminary Results of NGS Gene Panel Test Using NSCLC Sputum Cytology and Therapeutic Effect Using Corresponding Molecular-Targeted Drugs
Source: Genes (Basel). 2022 May 2;13(5):812. doi: 10.3390/genes13050812 (PMC9141607; doi:10.3390/genes13050812)
Supplement: Supplementary file 1 [file genes-13-00812-s001.zip › genes-1695408-supplementary.pdf]

Supplementary Materials

Table S1. Sequencing read depth and QC metrics of NGS result.

		Fastq quality	Amplification Bias (coefficient of variation of module1 depths)	average amplicon depths of DNA module1 region				
Identifier	SampleName	Q30_fastq	CV_module1	BRAF_e151_	KRAS_e021_	EGFR_e191_	EGFR_e201_	EGFR_e211_
SM-135	Case1_Sputum	99.62	0.117	12462.9	15433.3	13561.6	14771.7	16933.2
SM-136	Case1_TBB brush	99.64	0.088	12968.7	15304.7	13923.5	15905.6	15878.8
SM-226	Case2_Sputum	99.64	0.153	18488.5	27773.6	25230	21684	22391.5
SM-227	Case2_TBB brush	99.53	0.161	18761.9	27684.7	21526.4	24527.8	27511.4
SM-261	Case3_Sputum	99.66	0.1	7827.6	9802.8	7902.6	8503.8	9292.9
SM-262	Case3_TBB brush	99.73	0.14	6224.5	8936.8	8114.5	6857.7	7800.9

		Amplification Bias (coefficient of variation of module2	average amplicon depths of DNA module1 region							
Identifier	SampleName	CV_module2	EGFR_e181_depth	EGFR_e202_depth	EGFR_e212_depth	ERBB2_e081_depth	ERBB2_e171_depth	ERBB2_e201_depth	MET_e141_depth	MET_e142_depth
SM-135	Case1_Sputum	0.381	2816	6783.8	2873	5821.8	6703	8386.5	9420	7576
SM-136	Case1_TBB brush	0.576	3205	7858.9	2946	5682	10930.9	10656.7	6135	1510
SM-226	Case2_Sputum	0.48	8742.9	12908.9	10835.8	12462.9	19512.8	20088.3	31411.5	10251
SM-227	Case2_TBB brush	0.345	8796.9	12884.7	12119.9	14665.9	16476.7	15136.2	27004.4	18336.7
SM-261	Case3_Sputum	0.31	3763.9	7051.9	7872.9	5901.6	10386.8	10044.6	11236.9	9577.9
SM-262	Case3_TBB brush	0.529	3849	5545	4569.9	4254	6543.9	5484.5	13069.8	3425
